# Reviewing population studies for forensic purposes: Dog mitochondrial DNA

**DOI:** 10.3897/zookeys.365.5859

**Published:** 2013-12-30

**Authors:** Sophie Verscheure, Thierry Backeljau, Stijn Desmyter

**Affiliations:** 1 National Institute of Criminalistics and Criminology, Vilvoordsesteenweg 100, B-1120, Brussels, Belgium; 2 University of Antwerp (Evolutionary Ecology Group), Groenenborgerlaan 171, B-2020, Antwerp, Belgium; 3 Royal Belgian Institute of Natural Sciences (OD “Taxonomy and Phylogeny” and JEMU), Vautierstraat 29, B-1000, Brussels, Belgium

**Keywords:** Forensics, Mitochondrial DNA, Dog, Random match probability, Population study, Sampling strategy

## Abstract

The identification of dog hair through mtDNA analysis has become increasingly important in the last 15 years, as it can provide associative evidence connecting victims and suspects. The evidential value of an mtDNA match between dog hair and its potential donor is determined by the random match probability of the haplotype. This probability is based on the haplotype’s population frequency estimate. Consequently, implementing a population study representative of the population relevant to the forensic case is vital to the correct evaluation of the evidence. This paper reviews numerous published dog mtDNA studies and shows that many of these studies vary widely in sampling strategies and data quality. Therefore, several features influencing the representativeness of a population sample are discussed. Moreover, recommendations are provided on how to set up a dog mtDNA population study and how to decide whether or not to include published data. This review emphasizes the need for improved dog mtDNA population data for forensic purposes, including targeting the entire mitochondrial genome. In particular, the creation of a publicly available database of qualitative dog mtDNA population studies would improve the genetic analysis of dog traces in forensic casework.

## Introduction

Dogs (*Canis lupus familiaris*) are common and widespread in human society and hence, dog trace material is frequently encountered in forensic casework. Usually, this trace material involves hair, which is easily dispersed either through immediate contact with a dog or indirectly via an intermediate carrier, thus leaving a signature of the dog. Consequently, determining whether a particular dog could have donated the hair found at a crime scene may provide associative evidence (dis)connecting victims and suspects. For example, dog hairs could have been transferred from a victim’s clothes to the trunk of a perpetrator’s car during transportation of a body. Linking these hairs to the victim’s dog could connect the suspect to the crime.

**Figure 1.**
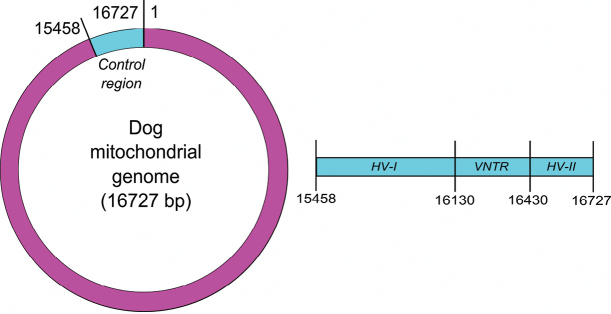
Position of the control region and its subregions within the Kim et al. (1998) reference dog mitochondrial genome.

Most dog hairs collected at crime scenes are naturally shed and are in the telogen phase. As such, because they contain only limited amounts of, usually degraded, nuclear DNA (nDNA), they are ill suited for nDNA analysis. Conversely, mainly as a result of its high copy number and much smaller size (Nass 1969, Bogenhagen and Clayton 1974), mitochondrial DNA (mtDNA) is quantitatively and qualitatively better preserved than nDNA in telogenic hairs and hence is far more suitable for analysis, as e.g. demonstrated in Gagneux et al. (1997) and Allen et al. (1998). To identify the mammal taxon that shed the hair, DNA barcoding can be applied through analysis of an mtDNA marker with little variation within and sufficient variation among taxa, often a part of cytochrome *b* or cytochrome *c* oxidase I in forensics (Linacre and Tobe 2011). On the other hand, in order to individualize dog hairs as accurately as possible, it is necessary to analyze mtDNA regions that show high variability among dogs and low intra-individual variation (heteroplasmy). As for human traces, this type of analysis focuses on the non-coding control region or D-loop (Wilson et al. 1993, Holland and Parsons 1999), which in dog mtDNA comprises about 1200 bp consisting of two hypervariable regions (HV-I and HV-II) separated by a Variable Number of Tandem Repeats (VNTR) region (Figure 1). This VNTR is a 10 bp tandem repeat with variable repeat numbers, both between and within individuals (length heteroplasmy). Because of its high level of length heteroplasmy, this repeat region is mostly not considered in forensics (Fridez et al. 1999). Several publications illustrate forensic casework involving control region analysis of dog traces, such as Savolainen and Lundeberg (1999), Schneider et al. (1999), Branicki et al. (2002), Aaspõllu and Kelve (2003), Halverson and Basten (2005) and Scharnhorst and Kanthaswamy (2011).

In general, mtDNA is maternally inherited (Sato and Sato 2013). In theory, this means that all dogs sharing a maternal line have the same mtDNA haplotype barring mutations. Hence, a match between the mtDNA of the dog hair found at a crime scene and that of a dog suspected of donating the trace, may be due to either of three possibilities: (1) the dog hair from the crime scene is from the suspected donor, (2) the hair from the crime scene is from a dog of the same maternal lineage as the suspected donor, (3) the mtDNA from the crime scene is by coincidence identical to that of the suspected donor. In order to assess the evidential weight of a match under the last scenario, one must calculate the haplotype’s random match probability, the probability that within a given population two randomly selected dogs will share the same haplotype by chance (Holland and Parsons 1999).

The random match probability is determined by the frequency estimate of the haplotype in the population of interest. The more common a haplotype, the higher is the probability that two dogs share this haplotype by chance, thus decreasing the evidential value of a match with this mtDNA type. Consequently, this sort of forensic applications requires the accurate estimation of haplotype frequencies in a population relevant to the criminal case.

The goal of this publication is to draw people’s attention to the importance of implementing a dog mtDNA population study representative of the population of interest in a forensic case. It will provide an overview of the most important issues to keep in mind both when performing a population study of your own, as well as when considering to use published mtDNA data. First of all, sampling strategy characteristics are discussed such as sample size, maternal relatedness, breed status of the sampled dogs, and their geographic origin. Next, the importance of the quality of the sequence data is emphasized. In addition, the need to expand the sequenced DNA fragment in dog mtDNA studies is illustrated. Finally, the advantages of, and the criteria for, the assembly of an international, publicly available dog mtDNA population database of the highest quality, are pinpointed.

## Estimating population frequencies of dog mtDNA haplotypes for forensic purposes

### Background

The accuracy of haplotype frequency estimates almost entirely depends on the characteristics of the population sample that is used to represent the relevant population, i.e. the population to which the donor of the trace is supposed to belong. Hence, biased population samples may lead to haplotype frequency estimates that diverge from the true population values.

To explore the impact of biased reference population samples in dog studies, we relied on current practices in human mtDNA population analyses and data derived from a selection of papers on haplotype variation in the dog mtDNA control region or the entire mitochondrial genome (mtGenome). Table 1 summarizes the main characteristics of the 58 dog studies used in this review. It includes studies with forensic aims, but also phylogenetic population and breed studies.

**Table 1.** Overview of the characteristics of sampling and sequence analysis in 58 canine mtDNA studies. Number of dogs sampled and, when specified in the publication, the number of dog breeds and mixed-breed, feral or village dogs in the sample; Origin of sample: new or extracted from previous studies as a comparison or to supplement the population sample (see reference numbers, except unpublished data by van Ash et al. (59); Koop et al. (60) and Shahid et al. (61)); Sampling region (or the geographic region of origin of included dog breeds if unclear from publication); Intention to avoid the inclusion of maternal relatives; GenBank accession numbers of new data are stated when applicable; un, unknown; s, skeletal remains of various age; ±, when variable, all sequences extracted from GenBank or the publication have this region in common; <, selected from this number of dogs from the same publication; * Larger region is mentioned in the publication, but only this part is available; Characteristics can differ from what is stated in publication if potential clerical errors were adapted, e.g. **publication states 246 instead of 233 as the sequences from reference 22 were included twice.

Publication			Sample						Sequence analysis	
	Reference	Aim of study	# Dogs	# Breeds	# Mixed- breed	Origin of sample	Sampling region	Avoid relatives	mtDNA region	Availability of new sequence data
1	Rothuizen et al. (1995)	mtDNA variability study	11	11	0	new	the Netherlands	un	Repeat region*	Publication
2	Okumura et al. (1996)	Phylogenetic breed study	94	24	0	new	Japan	un	15458-16129, 16420-16727	D83599-D83613, D83616-D83638
3	Savolainen et al. (1997)	Forensic, Phylogenetic	102	52	0	new	Sweden	YES	15431-15687	Publication
		Population study								
4	Tsuda et al. (1997)	Phylogenetic population study	34	24	0	new	Japan, Korea, Mongolia, Indonesia	un	15458-16130*	AB007380-AB007403
5	Vilà et al. (1997)	Phylogenetic population study	140	67	5	new	un	un	15431-15687	AF005280-AF005295
			15	un	un	< 140 new			± 15393-16076, ± 16508-49	AF008143-AF008157, AF008168-AF008182
6	Kim et al. (1998)	First complete dog mtGenome	1	1	0	new	Korea	YES	1-16727	U96639
7	Okumura et al. (1999)	Phylogenetic breed study	74s	un	un	new	Japan	un	15483-15679	AB031089-AB031107
			84	un	un	new				
			94	24	0	2			15458-16129, 16420-16727	
8	Vilà et al. (1999)	Phylogenetic breed study	19	1	0	new	US, Mexico	YES	15431-15687*	Publication
			140	67	5	5	un		15431-15687	
9	Randi et al. (2000)	Phylogenetic population study	41	30	0	new	Switzerland	un	15458-16000	AF115704-AF115718
			9	0	9	new	Italy			
10	Kim et al. (2001)	Phylogenetic breed study	25	11	0	new	Korea	NO	15622-16030	AF064569-AF064579, AF064581-AF064585
11	Branicki et al. (2002)	Forensic population study	12	11	1	new	Poland	un	15431-15687	AF345977-AF345982
12	Savolainen et al. (2002)	Phylogenetic population study	526	un	un	new	Europe, Asia, Africa, Arctic America	un	± 15458-16039	AF531654-AF531741
			128			2, 4				
13	Takahasi et al. (2002)	Inheritable disorder study	365	49	un	new	Japan	un	15458-16055	AB055010-AB055055
14	Valière et al. (2003)	Phylogenetic population study	50	un	un	new	France, Switzerland	un	± 15519-15746	AF487730-AF487735 (excl. AF487732),
			15	un	un	new	France, Portugal			AF487747-AF487751, AF338772-AF338788
15	Wetton et al. (2003)	Forensic population study	105	un	un	new	UK	un	15431-16030	AY928903-AY928932
			246			2, 3, 9	Japan, Switzerland, Italy, Sweden		± 15458-15687	
16	Pereira et al. 2004	Catalogue of published datasets	58	1	0	59	Portugal	un	15458-16039	Publication
			1089	un	un	2, 4, 10, 12, 13, 15	Europe, Asia, Africa, Arctic America		± 15622-16030	
17	Savolainen et al. (2004)	Phylogenetic population study	22	un	un	new	SE-Asia, India	un	15458-16039	AY660647-AY660650
			19s	un	un	new	Polynesia		15458-15720	Publication
			654	un	un	2, 4, 12	Europe, Asia, Africa, Arctic America		± 15458-16039	
18	Sharma et al. (2004)	Phylogenetic population study	24	0	24	new	India	un	15443-15783	AY333727-AY333737
19	Angleby and Savolainen (2005)	Forensic, Phylogenetic	35	19	9	new	Germany	YES	15458-16039	AY656703-AY656710
			74	52	2	new	Europe			
		Population study	758	un	un	2, 4, 12, 15	Europe, Asia, Africa, Arctic America		± 15458-16030	
20	Halverson and Basten (2005)	Forensic population study	348	88	45	new	US	un	15431-16085	Not published
21	van Asch et al. (2005)	Phylogenetic breed study	143	4	0	new	Portugal	YES	15372-16083	Publication
			144	9	0	2, 4, 12, 13	Europe, Asia, Africa, Arctic America		± 15458-16030	
22	Björnerfeldt et al. (2006)	Phylogenetic population study	88	53	0	new	Sweden	un	part of HV-I	Not published
			14	13	0	< 88 new			1-16727	DQ480489-DQ480502
23	Pires et al. (2006)	Phylogenetic breed study	143	11	0	new	Portugal, Spain, Morocco	YES	15211-16096	AY706476-AY706524
			21	0	21	new	Portugal, Azores, Tunisia			
24	Ryabinina (2006)	Phylogenetic breed study	84	3	0	new	Russia	un	15458-15778	DQ403817-DQ403837
			20	2	0	12	Turkey		± 15458-16039	
25	Sundqvist et al. (2006)	Phylogenetic breed study	100	20	0	new	Sweden	un	15431-15687	Publication
26	Eichmann and Parson (2007)	Forensic population study	133	46	38	new	Austria	un	15458-16727	Publication
27	Gundry et al. (2007)	Forensic population study	61	41	0	new	US	un	15455-16727	AY240030-AY240157
		Forensic breed study	64	2	0	new				(excluding AY240073, AY240094, AY240155)
28	Baute et al. (2008)	Forensic population study	83	30	0	new	US	un	15595-15654	Publication
			159			27, 30				
29	Hassell et al. (2008)	Forensic population study	96	79	0	new	UK	un	15458-16039	Not published
		Forensic breed study	15	1	0	new			15458-16131, 16428-16727	
30	Himmelberger et al. (2008)	Forensic population study	36	11	20	new	US (California)	un	15456-16063	EF122413-EF122428
			22	un	un	60	un		15433-16139	AF098126-AF098147
			179			2, 4, 5, 6, 10, 27	Europe, Asia, North-America		± 15622-16030	
31	Parra et al. (2008)	Phylogenetic breed study	52	5	0	new	Spain	un	15458-16105	EF380216-EF380225
32	Baranowska et al. (2009)	Inheritable disorder study	7	1	0	new	Sweden	NO	1-16727	FJ817358-FJ817364
33	Boyko et al. (2009)	Phylogenetic population study	309	0	309	new	Egypt, Uganda, Namibia	YES	± 15454-16075	GQ375164-GQ375213
			17	0	17	new	US (mostly Puerto Rico)			
			un	un	un	12, 23	East-Asia, Africa		± 15458-16039	
34	Desmyter and Comblez (2009)	Forensic population study	117	60	24	new	Belgium	YES	15458-16130, 16431-16727	Not published
35	Koban et al. (2009)	Phylogenetic breed study	114	2	0	new	Turkey	YES	15458-16039	EF660078-EF660191
			un	un	un	12	Europe, Asia, Africa			
36	Pang et al. (2009)	Phylogenetic population study	907	un	un	new, 61	Old World, Arctic America	un	± 15458-16039	EU816456-EU816557
			669	un	un	2, 4, 6, 12, 22				
			135	un	un	< 907 + 669			1-15511, 15535-16039, 16551-16727	EU789638-EU789786
			34	un	un	6, 22, 61			1-16727	AY656737-AY656755
37	Webb and Allard 2009a	Forensic population study	427	139	118	new	US	YES	± 15458-16114, ± 16484-16727	EU223385-EU223811
			125			27			15455-16727	
38	Webb and Allard 2009b	Forensic population study	64	43	11	37	US	YES	± 1-16129, ± 16434-16727	EU408245-EU408308
			15	14	0	6, 22	Korea, Sweden		1-16727	
39	Muñoz-Fuentes et al. (2010)	Phylogenetic population study	29	un	un	new	Canada	un	15361-15785	FN298190-FN298218
40	Smalling et al. (2010)	Forensic population study	220	0	220	new	US	YES	15456-16063	FJ501174-FJ501203
			429			30, 37			± 15458-16063	
41	Ardalan et al. (2011)	Phylogenetic population study	325	un	un	new	Europe, SW-Asia	un	15458-16039	HQ261489, HQ452418-HQ452423,
			1576	un	un	2, 4, 6, 12, 22, 36, 61	Old World, Arctic America		± 15458-16039	HQ452432-HQ452433, HQ452466-HQ452477
42	Brown et al. (2011)	Phylogenetic population study	200	0	200	new	Middle East/SW-Asia	un	15482-15867	HQ287728-HQ287744
			231	0	231	new	SE-Asia			
			1576	un	un	2, 4, 6, 12, 22, 36, 61	Old World, Arctic America		± 15458-16039	
43	Castroviejo-Fisher et al. (2011)	Phylogenetic population study	371	0	371	new	the Americas	un	± 15491-15755	HQ126702-HQ127072
			29	un	un	39				
44	Klütsch et al. 2011a	Phylogenetic population study	280	33	0	new	Europe, Arctic America, East-Asia	YES	15458-16039	GQ896338-GQ896345
			234			36			± 15458-16039	
45	Klütsch et al. 2011b	Point heteroplasmy pedigree study	180	18	0	new	Europe, Arctic America, East-Asia	NO	15458-16039	Publication
			131	2	0	new				
46	Kropatsch et al. (2011)	Phylogenetic breed study	77	26	0	new	Germany	NO	15458-16124	Publication
			34	1	0	new				
47	Li et al. (2011)	Phylogenetic breed study	1	1	0	new	China	YES	1-16727	HM048871
			33	un	un	22, 32, 38, 61	Sweden, US			
48	Sindičić et al. (2011)	Forensic species ID, Phylogenetic population study	20	0	20	new	Croatia	un	15465-15744	GU324475-GU324486
49	Bekaert et al. (2012)	Validation of forensic analysis method	41	29	3	new	Belgium	un	± 15458-16092, ± 16474-16703	HM561524-HM561546, HQ845266-HQ845282
			550			27, 37	US		± 15458-16114, ± 16484-16727	
50	Chakirou et al. (2012)	Phylogenetic breed study	78	3	0	new	Romania	YES	± 15251-16068	HE687017-HE687019
51	Desmyter and Gijsbers (2012)	Forensic population study	208	60	68	new, 34	Belgium	YES	15458-16129, 16430-16727	HM560872-HM560932
			778			15, 26, 27, 37	UK, Austria, US		± 15458-16030	
		Forensic breed study	107	6	0	new	Belgium		15458-16129, 16430-16727	
			337	6	0	< 208 new, 13, 19, 26, 27, 37	Worldwide		± 15458-16039	
52	Głażewska et al. (2012)	Phylogenetic breed study	34	2	0	new	Poland	NO	15426-16085	HM007196-HM007200
53	, Głażewska and Prusak (2012)		un	un	un	GenBank	Worldwide			
54	Imes et al. (2012)	Forensic population study	100	98	0	new	US, Australia, Canada, Columbia, Uruguay	YES	± 1-16129, ± 16430-16727	JF342807-JF342906
			233**	un	un	6, 22, 36, 38, 61	Worldwide		± 1-15511; ± 15535-16039; ± 16551-16727	
55	Li and Zhang (2012)	Phylogenetic breed study	47	1	0	new	Tibet, surrounding areas	YES	± 582 bp of control region	Not published
			439	un	un	GenBank	Worldwide			
56	Oskarsson et al. (2012)	Phylogenetic population study	305	un	un	new	SE-Asia, E-Asia	YES	15458-16039	HQ452439-HQ452465
			350	un	un	4, 12, 36			± 15458-16039	
			1224	un	un	2, 4, 6, 12, 22, 36, 61	Old World, Arctic America			
			19s	un	un	17	Polynesia		15458-15720	
57	Brown et al. (2013)	Phylogenetic population study	20s	un	un	new	Alaska, Greenland	un	± 367 bp of HV-I	JX185397
			51	1	0	new	Arctic America		± 15580-16016	
			78	2	0	2, 12, 36, 44			± 15458-16039	
58	Suárez et al. (2013)	Phylogenetic breed study	324	5	0	new	Canary Islands	YES	15361-16086	Publication
			986	un	un	15, 26, 27, 34, 37, 51	UK, Austria, Belgium, US		± 15458-16030	

Dog mtDNA studies quite often do not meet the standards required for generating and publishing forensic human mtDNA population data. Briefly, these standards include: (1) providing a good documentation of the sampling strategy and a detailed description of the sampled individuals and the population, (2) avoiding sampling bias due to population substructure, (3) applying high quality mtDNA sequencing protocols and describing them clearly, (4) avoiding errors by handling and transferring data electronically, (5) performing quality checks of the generated data by e.g. haplogrouping or quasi-median network analysis and (6) making the full sequences publicly and electronically available preferably through either GenBank (Benson et al. 2013) or a forensic database such as EMPOP (Parson and Bandelt 2007, Parson and Dür 2007, Carracedo et al. 2010, Parson and Roewer 2010, Carracedo et al. 2013). These standards will be discussed here in relation to dog mtDNA studies.

### Sampling strategy and its reporting

Strategies to sample mtDNA from dog populations are rarely well documented. Hence, it is often not clear to what extent the population samples adequately represent the populations from which they were drawn. Not seldom, sampling efforts are indeed limited to “sampling by convenience”, i.e. relying on opportunistic sampling from locations as veterinary clinics and laboratories, dog shows, training schools and animal shelters. Obviously, it can be doubted whether these sampling locations are representative random samples of the “free” living relevant dog community (Parson and Bandelt 2007). Moreover, the types of sampling locations are often not even specified. In addition, basic information on the sampled dogs is often rudimentary, as many studies do not mention (1) how many dogs are mixed-breed or purebred, (2) to which breeds the dogs belong and/or (3) whether the geographic information provided refers to the region of origin of the breeds or to the actual region where the dogs were sampled (Table 1).

Several publications have provided recommendations on population sampling strategies for both dog and human mtDNA in forensics (Parson and Bandelt 2007, Pereira et al. 2010, Webb and Allard 2010, Linacre et al. 2011, Scharnhorst and Kanthaswamy 2011). Although more detailed guidelines are lacking, the main issue with sampling a population in a representative manner is to avoid over- and underestimating haplotype frequencies. Sampling bias causes regarding the number and features of sampled individuals will be discussed in relation to dog mtDNA.

### Sample size

Using a random subsampling method (Pereira et al. 2004a), Webb and Allard (2010) assessed the influence of increased sample size on the distribution of haplotype frequency estimates in dog mtDNA population samples. They predicted that adding another 100 dogs to sample sizes of less than 650 dogs for HV-I and 750 dogs for HV-I and -II increases estimates of e.g. haplotype number and exclusion probability (i.e. the probability that two randomly chosen dogs from a sample have different haplotypes) with ≥ 5%. Table 1 shows that unless data are pooled, the majority of forensic dog control region population studies have rather small sample sizes of about 100 or fewer dogs.

Generally, the number of observed haplotypes increases with sample size (Table 2), while the proportion of rare haplotypes (i.e. encountered only once or twice) goes down. Consequently, exclusion probability largely remains the same with sample size expansion (Webb and Allard 2010) (Table 2). Under-sampling the population particularly affects the frequencies of haplotypes that remain rare while increasing sample size (Holland and Parsons 1999). This overestimation of rare haplotypes is illustrated when comparing nine forensic dog mtDNA studies. Many of the haplotypes with the highest frequencies in population samples of ≤ 100 dogs, have lower frequencies in larger sized studies (Table 2). For example, haplotype C5 occurs in 4.9% of the 61 dogs in the Gundry et al. (2007) study, while its frequency estimate is maximum 0.5% in other US studies in Table 2. Limited sample size thus tends to overestimate haplotype frequencies, which decreases the evidential value of an mtDNA match. Since overestimations do not inflate the risk of incriminating a false suspect, under-sampling can be deemed a conservative error (Salas et al. 2007).

**Table 2.** Comparison of haplotype number, P_E_ and haplotypes with the 10 highest frequencies in selected dog mtDNA studies. Exclusion probability (P_E_) is based on the part of the control region studied in the publication (further details on exact region in Table 1) excluding the repeat region; characteristics can differ from publication if potential clerical errors were adapted; the 3 universally most frequent haplotypes are in bold (**A11**, **B1** and **A17**); (×) US and a minority from Australia, Canada, Uruguay and Columbia; (××) Haplotype names are analogous to Savolainen et al. 2002, Savolainen et al. 2004, Angleby and Savolainen 2005, Pang et al. 2009, Klütsch et al. 2011a, Ardalan et al. 2011 and Oskarsson et al. 2012, or are in italic when unavailable and publication name was used; (×××) Haplotypes are based on 15458-16039 except for Wetton et al. 2003 (15458-16030). Only the Kim et al. (1998) reference nucleotide was considered in case of a heteroplasmic site.

	Population studies for forensic purposes																		Breed studies for forensic or phylogenetic purposes					
	Europe								US															
	Wetton et al. 2003		Angleby and Savolainen 2005		Eichmann and Parson 2007		Desmyter and Gijsbers 2012		Gundry et al. 2007		Himmelberger et al. 2008		Webb and Allard 2009a		Smalling et al. 2010		Imes et al. 2012		Okumura et al. 1996		Gundry et al. 2007		Suárez et al. 2013	
Sampling region	UK		Germany		Austria		Belgium		US		US		US		US		US (×)		Japan		US		Canary Islands	
Studied part of control region (CR)	HV-I		HV-I		entire CR		HV-I+II		entire CR		HV-I		HV-I+II		HV-I		HV-I		HV-I+II		entire CR		HV-I	
# Dogs (# breeds/# mixed-breed)	105(un/un)		35(19/9)		133 (46/38)		208 (60/68)		61(41/0)		36(11/20)		552 (139/118)		649		100(98/0)		94(24/0)		64(2/0)		324(5/0)	
# Haplotypes	31		13		40		58		32		16		104		71		34		38		13		16	
Exclusion probability	0.93		0.86		0.93		0.92		0.93		0.89		0.96		0.92		0.91		0.93		0.8		0.86	
Haplotypes with 10 highest frequency estimates (%) (××) (×××)	**B1**	14.2	**A11**	28.6	**A11**	18.0	**B1**	16.8	**A11**	14.8	**B1**	16.7	**B1**	15.6	**B1**	18.0	**B1**	17.0	A18	13.8	A33	29.7	**B1**	25.9
	A18	11.4	**A17**	14.3	**A17**	12.0	**A11**	15.4	**B1**	13.1	**A11**	13.9	**A17**	10.9	**A11**	12.6	**A11**	15.0	A68	10.6	A16	28.1	**A17**	14.5
	**A17**	10.5	**B1**	14.3	**B1**	12.0	**A17**	15.4	A18	9.8	A16	13.9	**A11**	10.3	**A17**	11.7	A18	14.0	C3	10.6	**B1**	26.6	A20	14.2
	A2	8.6	C3	8.6	A19	8.3	A19	6.7	**A17**	6.6	**A17**	13.9	A18	9.2	A18	11.1	**A17**	8.0	**A17**	9.6	A5	6.3	B6	14.2
	**A11**	8.6	A2	5.7	A2	6.8	A18	5.8	C3	4.9	A18	11.1	A16	6.7	A16	6.2	A2	7.0	B14	8.5	*Gundry_24*	6.3	A19	8.3
Haplotypes with 10 highest frequency estimates (%) (××) (×××)	A19	5.7	A19	5.7	A18	6.8	A16	4.8	C5	4.9	A1	2.8	A33	3.4	A19	3.1	A22	4.0	A19	4.3	**A11**	1.6	**A11**	6.5
	A16	4.8	B6	5.7	A16	3.8	A22	3.8	A1	3.3	A15	2.8	C3	3.1	C3	3.1	A5	3.0	A2	3.2	**A17**	1.6	A22	4.9
	A20	3.8	A5	2.9	A1	3.0	A2	2.4	A2	3.3	A22	2.8	A2	2.5	A2	2.5	A16	3.0	**A11**	3.2			*A17+*	2.8
	A26	2.9	A20	2.9	A153	3.0	C1	2.4	A64	3.3	A26	2.8	A19	2.4	A5	1.7	A167*	3.0	**B1**	3.2			A18	2.8
	A1	2.9	A33	2.9	A22	2.3	C2	2.4	C2	3.3	A28	2.8	A5	2.0	A22	1.7	A19	2.0	A29	2.1			A33	2.5
	C1	2.9	A44	2.9	A26	2.3			*Gundry_31*	3.3	A29	2.8			C2	1.7	A24	2.0	A70	2.1				
			A70	2.9	A33	2.3					A64	2.8							A72	2.1				
			A82	2.9	C1	2.3					A140	2.8							B12	2.1				
											A156	2.8							C1	2.1				
											B3	2.8												
											C3	2.8												

### Maternal relationships

A randomized population sample for forensics should be allowed to include relatives if it is supposed to be unbiased (Brenner 2010). However, many population samples are assembled by convenience and could therefore contain more maternal relatives than expected from a randomized sample (Bodner et al. 2011).

**Figure 2.**
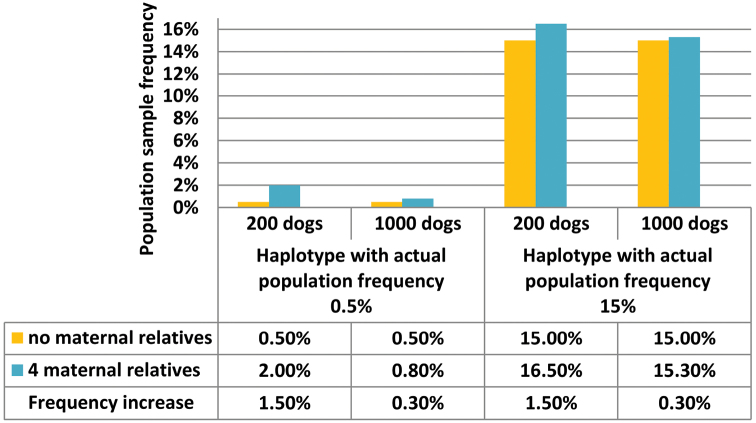
Impact of including maternally related dogs in population samples of 200 versus 1000 dogs on the estimation of the frequencies of rare haplotypes.

The impact of a biased inclusion of maternal relatives in a forensic population study is rarely addressed, but generally decreases the genetic diversity of the population sample (Webb and Allard 2009a, Webb and Allard 2010). In small population samples, it particularly affects the risk of over-representing rare haplotypes (Bodner et al. 2011). By way of example, Figure 2 demonstrates that the impact of including 4 maternally related dogs is 5 times higher in a sample of 200 compared to 1000 dogs. In the smaller sample, the biased inclusion of 4 maternal relatives sharing a rare haplotype even causes its population sample frequency to be quadrupled. Moreover, even when observed only once in a population study, a haplotype that is rare in a population is already typically overrepresented in that sample (Holland and Parsons 1999). Therefore, oversampling maternal relatives should be avoided.

Although not specifically mentioned as a criterion for human mtDNA data in the international EMPOP database (Parson and Dür 2007), published population studies submitted to EMPOP do state that, as far as could be ascertained, the sampled individuals are unrelated. Examples are Brandstätter et al. (2007), Irwin et al. (2007), Saunier et al. (2009) and Prieto et al. (2011). Moreover, maternal relatives are removed in database updates. For human mtDNA population studies, it has been recommended to assess familial relationships by screening both available donor information and nDNA variation using microsatellites (Bodner et al. 2011).

For dog studies there is no consistent practice in dealing with maternal relationships in the population samples. Only about half of the 58 dog mtDNA studies in Table 1 mention whether or not they had the intention to avoid maternal relatives. Obviously, the usefulness of studies that do not provide this information may be doubtful in forensics. How maternal relationships were assessed is often not specified either, but it usually involves collecting information about the dogs from their owners. These background records can be used to verify whether dogs sharing a haplotype are e.g. from the same breed or whether their places of residence or those of their parents coincide (Webb and Allard 2009a, Desmyter and Gijsbers 2012). However, since dogs can have lots of offspring, there could be many maternal relatives, and these may be hard to track. Also, information provided by owners is not necessarily reliable (Webb and Allard 2009a) or available, and even registered pedigree records can be erroneous or incomplete (Kropatsch et al. 2011).

### Purebred versus mixed-breed dogs

Another characteristic that can affect the haplotype frequency distribution in a population sample is potential population substructure due to the existence of dog breeds. Indeed, although generally mtDNA does not allow dogs to be grouped into their respective breeds (Okumura et al. 1996, Savolainen et al. 1997, Tsuda et al. 1997, Vilà et al. 1997, Kim et al. 2001, Wetton et al. 2003, Angleby and Savolainen 2005, van Asch et al. 2005, Pires et al. 2006, Sundqvist et al. 2006, Eichmann and Parson 2007, Gundry et al. 2007, Himmelberger et al. 2008, Parra et al. 2008, Desmyter and Comblez 2009, Kropatsch et al. 2011, Bekaert et al. 2012, Desmyter and Gijsbers 2012, Suárez et al. 2013), haplotype frequencies can differ between breeds, as well as between specific breeds and the entire dog mtDNA gene pool (Savolainen et al. 1997, Vilà et al. 1999, Angleby and Savolainen 2005, van Asch et al. 2005, Pires et al. 2006, Ryabinina 2006, Eichmann and Parson 2007, Gundry et al. 2007, Hassell et al. 2008, Himmelberger et al. 2008, Parra et al. 2008, Koban et al. 2009, Webb and Allard 2009a, Kropatsch et al. 2011, Desmyter and Gijsbers 2012, Brown et al. 2013, Suárez et al. 2013). Because of the overrepresentation of certain haplotypes in specific breeds in comparison to the general dog population, a dog trace mtDNA type might provide an indication about the breed(s) to which it may belong. However, such information should be used with caution and police investigations should not only focus on the more likely breed (Angleby and Savolainen 2005, Hassell et al. 2008, Desmyter and Gijsbers 2012).

Obviously, the over- and underrepresentation of particular breeds in a population sample compared to the population from which the sample is drawn, may bias haplotype frequency estimates (Desmyter and Gijsbers 2012). Therefore, in theory, dogs should be randomly sampled in order to correctly represent the breed composition of the population of interest (Scharnhorst and Kanthaswamy 2011). In addition, it is recommended that population samples reflect the actual proportions of mixed-breed versus purebred dogs in the population. However, mixed-breed dogs are often under-represented in population studies (Smalling et al. 2010) and many population studies even only include purebred dogs (Table 1). Moreover, since most samples are collected by convenience, deviations from the actual breed composition of the population and overrepresentation of rare dog breeds are to be expected. This should be taken into account when using this sort of data in forensic casework (Eichmann and Parson 2007).

Against this background, Savolainen et al. (1997) attempted to adjust the number of dogs per breed in their population sample, so as to more accurately reflect the countrywide breed composition in Sweden. Later, Angleby and Savolainen (2005) stated that the exclusion probability of population samples containing dogs of the 20 most common breeds in Sweden represent the Swedish population more accurately than population samples containing the 100 most common breeds. Still, the number of dogs per breed and the overall number of breeds in a sample can be overestimated, since these data largely depend on the owner’s subjective opinion (Himmelberger et al. 2008, Webb and Allard 2009a).

Some authors have indicated that population studies specific for single breeds may be forensically relevant in the rare event that the breed of the dog that donated the crime scene trace is known, for example by eye-witness reports (Savolainen et al. 1997, Wetton et al. 2003, Desmyter and Gijsbers 2012). Obviously, the evidential value of an mtDNA match can be quite different when based on a general rather than a breed-specific population study. Studies focused on specific breeds have been published, mostly aiming at verifying the accuracy of pedigree records and tracing its population genetic features (e.g. demographic history, region of origin, hybridization events, etc.). Examples are Vilà et al. (1999), van Asch et al. (2005), Kropatsch et al. (2011) and Suárez et al. (2013).

Including pedigree data can improve intra-breed mtDNA diversity studies. In theory, an appropriate selection of representative individuals from existing maternal lines from pedigrees allows to capture all mtDNA haplotypes of a breed within a population while minimizing the amount of laboratory work. The frequencies of these haplotypes can be estimated from the numbers of offspring in each maternal line in the breed population (Głażewska et al. 2013). Of course, to this end pedigree records need to be accurate and complete (Głażewska et al. 2013). Unfortunately, this is not always the case, as has been shown in e.g. Weimaraner dogs (Kropatsch et al. 2011).

Analyzing the haplotype frequency distribution within breeds can also give insight into differences between published population studies.An example of the impact of breed associated sample bias was given by Desmyter and Gijsbers (2012). These authors noted that the US population sample of Webb and Allard (2009a) included 64 dogs of two Retriever breeds from Gundry et al. (2007). This could have biased the frequency estimates of haplotypes A16 and A33 in the US sample, since these haplotypes are very common in Retrievers (Desmyter and Gijsbers 2012). Additionally, mtDNA studies focusing on specific breeds rather than on entire populations, clearly show lower amounts of variation (expressed in terms of exclusion probability) than population studies from similar geographical regions (Table 2). Also, the sets of haplotypes with the ten highest frequencies can be quite different (Table 2). In order to compensate for purebred related biases, Himmelberger et al. (2008) increased the number of mixed-breed dogs in their US population sample and claimed that in this way their sample was more representative than previous US dog mtDNA population samples. However, their sample still showed an unusually high frequency of haplotype A16 (Table 2), most probably because their sample was limited to only 36 dogs, 13 of which were either purebred or mixed-breed Retrievers.

### Geographic origin

To evaluate the significance of a haplotype match between a dog trace and its suspected donor, a population sample should reliably reflect the population to which the donor of the trace is supposed to belong. As such, one might wonder about the importance of the geographic origin of the sampled dogs in a sampling strategy.

Probably the most important macrogeographic issue to consider in dog studies, is the fact that dog populations in Southeast Asia show almost the entire dog mtDNA diversity, while elsewhere in the world only parts of this diversity is present (Savolainen et al. 2002, Pang et al. 2009, Ardalan et al. 2011, Brown et al. 2011). This suggests that SE Asia is the region where dogs were first domesticated and from where domesticated dogs were spread throughout the rest of the world (Savolainen et al. 2002, Pang et al. 2009). Another noticeable macrogeographic structuring in dog mtDNA is that haplotype group d1 is almost exclusively found in Scandinavian and Finnish breeds, in which sometimes over 50% of the dogs have a d1 haplotype (Klütsch et al. 2011a). Obviously, this sort of macrogeographic mtDNA differentiation should be considered in population sampling, since oversampling dog breeds of SE Asian or Scandinavian/Finnish origin in local population samples elsewhere in the world can bias haplotype frequency estimates. For example, Angleby and Savolainen (2005) demonstrated that dogs of East Asian origin in Europe carried a number of haplotypes that are absent in native European breeds. Moreover, the frequencies of globally common haplotypes differ between Asian and European samples (Angleby and Savolainen 2005). This is also illustrated by the composition and frequency distribution of the most common dog haplotypes in the breed study from Japan by Okumura et al. (1996) and those in the forensic population studies from Europe and the US (Table 2).

### Quality of nucleotide sequence data

The description of haplotypes is a source of error and confusion when comparing population studies. Typically, haplotypes are aligned to a reference sequence using software supplemented with annotation rules in order to record them unambiguously as an alpha-numeric code. This code is a shortened annotation of the sequence string, consisting of differences to the reference sequence. For example, the HV-I alpha-numeric code of haplotype A11 is 15639A, 15814T and 16025C (Angleby and Savolainen 2005). Analogous to human mtDNA analyses, Pereira et al. (2004b) recommended to set the L-strand of the first published complete dog mtGenome (Kim et al. 1998) as the reference standard. In order to identify different haplotypes and enable their comparison, Pereira et al. (2004b) listed a number of rules to align sequences to the Kim et al. reference (1998) and to unambiguously record polymorphisms. These rules are based on those for human mtDNA (Carracedo et al. 2000, Wilson et al. 2002a, Wilson et al. 2002b). Length heteroplasmy in the VNTR region of the dog’s mtDNA control region complicates the numbering system of the nucleotide positions. To simplify this, Pereira et al. (2004b) decided that numbering the nucleotide positions after this repeat region should start at position 16430 regardless of the number of repeats (Figure 1). Nevertheless, even with a standard reference haplotype, a numbering system and annotation rules, variation can still be miscoded, such as for the polyC-polyT-polyC region from position 16661 to 16674 in HV-II (Table 3).

**Table 3.** Illustration of different annotations for the HV-II polyC-polyT-polyC haplotype with 6 C’s, 8 T’s and 2 C’s. Annotation (1) was used by Gundry et al. (2007), while Eichmann and Parson (2007) and Desmyter and Gijsbers (2012) applied annotation (2) because of different alignments to the Kim et al. (1998) reference sequence of 3C8T3C.

**#C#T#C**	**16661**	**16662**	**16663**	**16663.1**	**16663.2**	**16663.3**	**16664**	**16665**	**16666**	**16667**	**16668**	**16669**	**16670**	**16671**	**16672**	**16673**	**16674**
**3C8T3C**	**C**	**C**	**C**	-	-	-	**T**	**T**	**T**	**T**	**T**	**T**	**T**	**T**	**C**	**C**	**C**
**6C8T2C (1)**	C	C	C	C	C	-	C	T	T	T	T	T	T	T	T	C	C
**6C8T2C (2)**	C	C	C	C	C	C	T	T	T	T	T	T	T	T	C	C	-

Haplotypes can also be denoted by names. However, it is not good practice to provide only haplotype names in publications, like e.g. Sundqvist et al. (2006) did. This introduces ambiguities if the same names are used elsewhere for other haplotypes. For the same reason, it is ill advised to use haplotype names that differ from GenBank entries, as was done by e.g. Smalling et al. (2010). The haplotype names established by Savolainen et al. (2002), Savolainen et al. (2004) and Angleby and Savolainen (2005), were expanded by Pang et al. (2009) and Webb and Allard (2009a), such that they both used the same names for different new haplotypes. Since then, names of new haplotypes often overlap between publications that are building further onto the names of both of these publications, e.g. Smalling et al. (2010), Ardalan et al. (2011), Klütsch et al. (2011a) and Imes et al. (2012). In addition, applying previously published haplotype names can be difficult because the analyzed mtDNA region may differ (Pereira et al. 2004b).

Mistakes occur relatively often while copying and editing sequence data. Therefore, guidelines have been published to minimize making these clerical errors and to detect them more easily (Bandelt et al. 2001, Bandelt et al. 2004, Yao et al. 2004, Salas et al. 2005). For example, alpha-numeric codes presented in the form of a matrix-based dot table are particularly error-prone and difficult to read (Parson and Bandelt 2007, Parson and Roewer 2010). In practice, several clerical errors have been observed in dog mtDNA studies. For example, alignment with the Kim et al. (1998) reference sequence of the GenBank entries corresponding to the HV-I codes in Table 2 of Imes et al. (2012), revealed several inconsistencies. A deletion at position 15932 in many haplotypes in Table 2 of Imes et al. (2012) cannot be observed in most of the GenBank entries. As such, this deletion defined two artificial haplotypes. Furthermore, Table 2 of Imes et al. (2012) did not include two haplotypes deposited in GenBank, while variant base 15665C was not recorded for haplotype A170*.

As more mtGenome data are generated, coding regions SNPs are encountered that appear to be characteristic for particular control region haplotypes and haplogroups (Verscheure, unpublished data). Such SNPs can help to indicate potential sequence or clerical errors. For example, the control region sequence of mtGenome haplotype A169* (A11 after removal of the deletion at 15932) belongs to haplogroup A (Imes et al. 2012), but the SNPs in the rest of its mtGenome are typical for haplogroup B. This might be due to artificial recombination, caused by mixing up amplicons from different individuals, either during laboratory work or data editing (Bandelt et al. 2001, Bandelt et al. 2004). Similarly, the entire mtGenome sequence of Imes et al. (2012) haplotype A167* is more typical of haplogroup C than of haplogroup A.

As shown above, deposition of sequence data in GenBank provides an opportunity to verify sequence data quality. Unfortunately, in contrast to good practice, 15 of the 58 studies reviewed here did not submit any sequence to GenBank, but only provided alpha-numeric codes or haplotype names. Moreover, several papers did not even disclose the haplotype sequences or their estimated population frequencies (Table 1). When studies did deposit sequences in GenBank, they did so either only for new haplotypes, for all observed haplotypes, or for all sampled dogs. These various practices may confound subsequent analyses. For example, Imes et al. (2012) extracted the mtGenomes of the same 14 dogs twice from GenBank, because these sequences were uploaded in GenBank both by Björnerfeldt et al. (2006) and Pang et al. (2009). Obviously, these duplicated datasets introduce bias in the estimation of mtGenome haplotype frequencies and mtDNA diversity in the Imes et al. (2012) study.

Dog mtDNA studies show a large variety of analysis methods as well. Consequently, the quality of these analyses might vary. Next to annotation issues, several sequence quality issues have been observed while reviewing dog mtDNA studies. For example, Webb and Allard (2009a) reported sequence reading difficulties in the HV-II region because of length heteroplasmy in the VNTR and the polyC-polyT-polyC region. Nevertheless, in about 190 sequences Webb and Allard (2009a) observed that positions 16430, 16431, 16432 and/or 16433 directly adjacent to the VNTR at the start of HV-II are deleted in comparison to the Kim et al. (1998) reference sequence. Since deletions have not been reported at these sites in any of the other reviewed studies, we suggest these should be considered missing data due to reading difficulties. Therefore, it is recommended to verify this issue using additional primers, other alignment software and visual inspection of the alignments. Webb and Allard (2009a) interpreted these sites as highly informative and counted sequences differing only at these positions (e.g. A18b and d) as different haplotypes. If these deletions indeed resulted from reading difficulties, then they artificially increased the haplotype number and exclusion probability. A second example is that about 65% of the mtGenome sequences deposited in GenBank by Imes et al. (2012) contain ambiguities outside the VNTR with up to 130 N’s per sequence and stretches of up to 110 adjacent N’s, i.e. ambiguous bases due to the presence of dye blobs (Imes et al. 2012). If such ambiguities occur at informative sites, then these sequence quality issues can affect the frequency estimates of mtGenome haplotypes and the SNPs that define them.

Thus, caution and proofreading is necessary for both new sequences and those extracted from papers and databases. Therefore, Berger et al. (2012) published a detailed workflow for generating high quality HV-I and -II data from dogs based on experience from human mtDNA analysis. For forensic mtDNA analysis, these and other authors recommend to sequence each position at least twice, preferably on both mtDNA strands, so as to minimize sequencing errors (Wilson et al. 1993, Carracedo et al. 2000, Tully et al. 2001, Parson and Bandelt 2007, Berger et al. 2012). Finally, when extracting sequences from GenBank, it is important to realize that quality control of a database entry relies on the submitting scientist. Hence, it is not surprising that the reliability of GenBank data has been questioned, such as by Harris (2003) and Yao et al. (2009).

### Exploring the entire mtGenome to improve discriminatory power

The majority of dogs have haplotypes that are frequent in most dog populations worldwide. As a result, even if there are many rare haplotypes, the discriminatory power of the dog mtDNA control region is limited (Savolainen et al. 1997, Wetton et al. 2003, Angleby and Savolainen 2005, Halverson and Basten 2005, Eichmann and Parson 2007, Gundry et al. 2007, Baute et al. 2008, Hassell et al. 2008, Himmelberger et al. 2008, Desmyter and Comblez 2009, Webb and Allard 2009a, Smalling et al. 2010, Desmyter and Gijsbers 2012, Imes et al. 2012). This is well illustrated by comparing the mtDNA characteristics of nine forensic population studies which all consider at least positions 15458 to 16030 in HV-I. Almost half of the sampled dogs have haplotypes B1, A11 or A17 with average population frequency estimates of 15.3%, 15.2% and 11.5%. In addition, many other frequent haplotypes are shared between samples (Table 2). Hence, dog mtDNA matches will often have limited forensic value.

Evidently, expanding the length of the surveyed sequence will increase the number of polymorphic sites and thus may improve the discriminatory power of the mtDNA control region in dogs. However, most population studies did not include HV-II and as such missed important variation that often allows splitting up HV-I haplotypes. Hence, sequencing at least both HV-I and HV-II is recommended for forensic population studies (Eichmann and Parson 2007, Gundry et al. 2007, Desmyter and Comblez 2009, Webb and Allard 2009a, Webb and Allard 2010, Desmyter and Gijsbers 2012, Imes et al. 2012).

A number of complete control region haplotypes still show high population frequencies. Therefore, it is advised to further increase the discriminatory power of dog mtDNA by surveying population samples for entire mtGenomes (Webb and Allard 2009a). This is indeed a trend in the last years with very promising results (Webb and Allard 2009b, Imes et al. 2012). However, the use of SNPs in the coding region in forensics will require many more mtGenome studies (Irwin et al. 2011).

### Population study versus database

Not every forensic laboratory has the resources to conduct large-scale population studies. As such, supplementing smaller, local samples with published data allows capturing more mtDNA variability. However, this practice may bias the haplotype frequency distribution in the pooled sample compared to the population of interest, because of (1) sample heterogeneity, (2) inconsistent sequence quality, (3) clerical errors and (4) the difficulty of sequence comparisons due to variation in sequence lengths, alignment procedures, and sequence annotation. Relying on a public dog mtDNA database instead of, or in addition to, published local population data may be a trustworthy alternative, provided that the sequences are carefully reviewed before inclusion in the database. As such, submitting population sample data to the database could be an obligatory quality check with which studies have to comply before they are published. This is often demanded for human mtDNA population data (Carracedo et al. 2010, Parson and Roewer 2010, Carracedo et al. 2013).

To establish a reliable dog mtDNA database, inspiration can be found in the European DNA profiling group (EDNAP) mtDNA population database (EMPOP) for human mtDNA haplotypes useful in forensic casework. EMPOP stresses the need for generating mtDNA sequence data of the highest quality (Parson et al. 2004, Parson and Dür 2007) and established guidelines to achieve this. Briefly, these guidelines recommend: (1) application of a high quality mtDNA determination method that covers the entire sequenced region at least twice, (2) electronic transfer and transcription of sequence results, (3) compliance to generally accepted alignment and annotation guidelines and (4) data verification through haplogrouping and quasi-median network analysis (Brandstätter et al. 2007, Parson and Dür 2007). Against this background, the interlaboratory study by van Asch et al. (2009) emphasized a similar need for such guidelines for dog mtDNA analyses.

Next to the need for high quality mtDNA population data from all around the world, three other important requirements for building a dog mtDNA database are discussed hereafter. Firstly, management by a central laboratory is indispensable to perform the quality assessment of submitted population samples, to maintain and update the database software and web portal, and to communicate about it to the users. After submission to EMPOP, this laboratory reviews the population sample data for errors by e.g. examining the raw sequence data and using quasi-median network analysis (Bandelt and Dür 2007, Parson and Dür 2007, Zimmermann et al. 2011). Indeed, allocating mtDNA sequences to specific haplogroups may indicate which mutations are expected and may help to detect potential artificial recombination (Bandelt et al. 2001, Bandelt et al. 2004, Bandelt et al. 2012). Additionally, EMPOP provides its users with software for network analysis that may point out potential errors within the data based on phylogenetic background information. Thorough phylogenetic knowledge of dog mtDNA haplotypes could allow the adaptation of such software for dog mtDNA population samples.

Secondly, the database should be searchable and provide tools for comparison of various mtDNA sequence ranges. EMPOP uses the SAM search engine, which translates the queried haplotype and all database entries into sequence strings that are more easily comparable than alpha-numeric codes. In this way, it avoids generating biased haplotype frequency estimates caused by alignment and annotation inconsistencies making that database entries remain undetected in a database search even if they are identical to the queried haplotype (Röck et al. 2011).

Finally, the database should sufficiently document background information on the specimens. This enables the selection of subsets of samples in the database relevant to a specific case, such as dogs from specific geographic regions, of particular breeds, etc. In casework, selection of a suitable dataset is vital to a correct evaluation of evidence. Weighing the evidence against several database subdivisions is recommended to consider which one provides the most appropriate and conservative estimate of a haplotype’s random match probability (Salas et al. 2007).

FidoSearch^TM^, a canine mtDNA database with search software, was developed for use in casework by the Institute of Pathology and Molecular Immunology in Porto, Portugal in collaboration with Mitotyping Technologies in Pennsylvania, USA (Melton et al. 2011). However, it is not publicly available and its data entries were assembled from GenBank. Hence, FidoSearch^TM^ is not an appropriate alternative for the creation of a publicly available, high quality and comprehensive dog mtDNA database.

## Conclusions

In order to meet forensic quality standards, a dog mtDNA population sample needs to be representative of the population of interest to the case. To this end, several recommendations can be made for performing and publishing a dog mtDNA population study for forensic purposes: (1) provide sufficiently detailed information on the population of interest, the sampling strategy and the sampled dogs, (2) include at least several hundred dogs in the population sample, (3) intend to avoid biased inclusion of maternal relatives, (4) use a population sample reflecting the dog population where the crime occurred, (5) the composition of the population sample in terms of purebred and mixed-breed dogs, groups of breeds of a particular geographic origin, and dogs belonging to specific breeds, should be proportional to the studied population, (6) apply a high quality and validated analytical methodology and run quality control steps to minimize the risk of errors during either laboratory work or data processing, (7) submit the haplotype sequence strings to a publicly available database such as GenBank and (8) follow the Pereira et al. (2004b) rules when converting haplotype sequences into alpha-numeric codes denoting differences in relation to the Kim et al. (1998) reference sequence. These recommendations also apply when supplementing your own data with published data. In addition, keep in mind that sequence files in a database such as GenBank do not provide raw sequence data and can hide ambiguous results.

All things considered, this review emphasizes the need for more forensically relevant, high quality dog mtDNA population studies. In addition, it stresses the need for a publicly available dog mtDNA population database that assembles easily comparable and thoroughly checked population data from all around the world. Finally, expanding mtDNA studies from the control region to the entire mtGenome is recommended to enhance the discriminatory power of forensic dog mtDNA analysis.
